# Transition and Transversion Mutations Are Biased towards GC in Transposons of *Chilo suppressalis* (Lepidoptera: Pyralidae)

**DOI:** 10.3390/genes7100072

**Published:** 2016-09-24

**Authors:** Guang-Hua Luo, Xiao-Huan Li, Zhao-Jun Han, Zhi-Chun Zhang, Qiong Yang, Hui-Fang Guo, Ji-Chao Fang

**Affiliations:** 1 Institute of Plant Protection, Jiangsu Academy of Agricultural Sciences, Nanjing 210014, China; xhli2012@163.com (X.-H.L.); zczhangjs@163.com (Z.-C.Z.); mlyangqiong@163.com (Q.Y.); guohfjaas@163.com (H.-F.G.); 2 College of Plant Protection, Nanjing Agricultural University, Nanjing 210095, China; zjhan57@126.com

**Keywords:** transposon, transition, transversion, indels, patterns

## Abstract

Transposons are often regulated by their hosts, and as a result, there are transposons with several mutations within their host organisms. To gain insight into the patterns of the variations, nucleotide substitutions and indels of transposons were analysed in *Chilo suppressalis* Walker. The *CsuPLE1.1* is a member of the *piggyBac*-like element (PLE) family, which belongs to the DNA transposons, and the *Csu-Ty3 * is a member of the Ty3/*gypsy* family, which belongs to the RNA transposons. Copies of *CsuPLE1.1* and *Csu-Ty3* were cloned separately from different *C. suppressalis* individuals, and then multiple sequence alignments were performed. There were numerous single-base substitutions in *CsuPLE1.1* and *Csu-Ty3*, but only a few insertion and deletion mutations. Similarly, in both transposons, the occurring frequencies of transitions were significantly higher than transversions (*p* ≤ 0.01). In the single-base substitutions, the most frequently occurring base changes were A→G and T→C in both types of transposons. Additionally, single-base substitution frequencies occurring at positions 1, 2 or 3 (pos1, pos2 or pos3) of a given codon in the element transposase were not significantly different. Both in *CsuPLE1.1* and *Csu-Ty3*, the patterns of nucleotide substitution had the same characteristics and nucleotide mutations were biased toward GC. This research provides a perspective on the understanding of transposon mutation patterns.

## 1. Introduction

Knowledge of the patterns and frequencies of substitution mutations in genomes is important in studying molecular evolution and understanding the molecular basis of substitution mutations [1,2,3]. Generally in evolutionary theory, mutations are considered random with respect to their adaptive values to the organism. However, it has long been recognized that this does not necessarily imply randomness in other respects. Many studies have indicated that some nucleotides are more mutable than others [4,5]. Biased patterns of nucleotide substitution are sources of non-randomness in the production of heritable variation [6]. Pseudogenes are genes that are not exposed to selective pressure or functional constraints, and hence are free to accumulate mutations in their sequences [5,7,8]. As a result, comparing the sequences of functional genes to their corresponding pseudogenes is a very powerful approach to reveal mutation patterns. Similarly, unconstrained sequences (non-long term repeat (LTR) retrotransposable elements) present in organisms that lack large numbers of pseudogenes can also be used in studying mutation pattern [6].

There are two intrinsic problems associated with pseudogenes that can potentially introduce bias in studies of mutation patterns [5]. First, when aligning sequences between a pseudogene and a functional gene, it is often difficult to distinguish whether a gap in the sequence is the result of a deletion or an insertion event. In a previous study, a large number of ambiguous indels were classified as ‘gaps’ rather than insertions or deletions [9]. The second problem is that sequence alignment and comparison should be performed between the pseudogene and the ancestral gene from which the pseudogene was derived. However, due to the evolution and/or variation in both the functional gene and the pseudogene, it is often difficult to determine whether the differences in the sequence alignment reflect the substitutions in the pseudogenes or in the functional genes.

Transposable elements (TEs) are mobile genetic DNA sequences, and they are found in the genomes of nearly all eukaryotes [10]. TEs are often regulated by their hosts, and as a result, there are a lot of mutations within TE sequences [11]. A given TE copy in a specific locus in the genome of a host can be easily obtained by flanking PCR. Based on the flanking PCR, TE copies can be determined and then multiple sequence alignments performed. Thus, the patterns of the variations in TE sequences can be determined.

In this paper, we describe a study on the sequence mutation patterns in a DNA transposon and a RNA transposon in *Chilo suppressalis*. We cloned *CsuPLE1.1* (GenBank accession No. JX294476), a member of the *piggyBac*-like element (PLE) family, a DNA transposon, and *Csu-Ty3* (GenBank accession No. KJ191261), a member of the Ty3/*gypsy* family, a RNA transposon [12,13]. For both transposons, a transposon copy was obtained from the same locus in different individuals, which were randomly selected from different field populations. Multiple alignments of *CsuPLE1.1* and *Csu-Ty3* sequences were performed, followed by an analysis of the patterns of nucleotide variation and the patterns of deduced amino acid variation. Many similar studies have focused on a huge number of sequences which were obtained from only one or a few individual samples based on high-throughput sequencing. This study focused on sequencing one locus in different individuals, from randomly selected field populations. This study might add new perspective in the efforts to understand transposon sequence mutations.

## 2. Material and Methods

### 2.1. Sample Collection and DNA Isolation

The *C. suppressalis* samples were randomly collected from 21 rice paddy field locations in China (Table S1), and stored at −80 °C until use. Genomic DNA (gDNA) was prepared using an AxyPrep DNA Extraction kit (Axygen Biosciences, Hangzhou, China) following the manufacturer’s protocol.

For *CsuPLE1.1*, a total of 84 individual samples were randomly selected from 21 field populations for this study (four individuals in each population) (Table S1). When multiple sequence alignments were performed, one *CsuPLE1.1* copy in one field population was selected and a total of 21 *CsuPLE1.1* copies were considered as one group (Dataset S1). Thus, there were four groups for performing alignments.

For *Csu-Ty3*, a total of 140 individual *C. suppressalis* were randomly selected from seven field populations (DY (Deyang), JJ (Jiangjin), JZ (Jingzhou), XY (Xiangyin), QC (Qichun), HX (Hexian) and YX (Yinxian)) (Table S1). Twenty individuals were selected in each population. Because of choosing fewer field populations, more *Csu-Ty3* sequence copies were taken for multiple sequence alignment. When multiple sequence alignments were performed, five *Csu-Ty3* copies in one field population were selected and a total of 35 *Csu-Ty3* copies were considered as one group (Dataset S2).

### 2.2. Determination of the CsuPLE1.1 Copy at the Same Locus in Different Individuals

**Figure 1:**
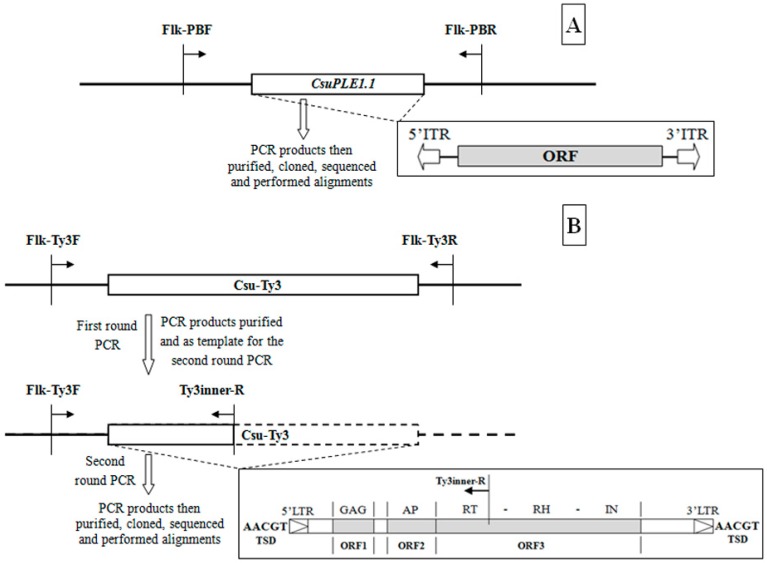
The strategy for cloning *CsuPLE1.1* and *Csu-Ty3* copy. The open bar represents the transposons *CsuPLE1.1* (**A**) or *Csu-Ty3* (**B**). The bold line represents the genome sequences of *C. suppressalis*. The left and right black arrowheads represent the primer pairs. The small pictures on the lower right (**A**,**B**) represent the transpsosn’s structure. The blank arrows represent the ITR (inverted terminal repeat). AACGT, is the TSDs (target site duplications). The ORF (open reading frame) encodes transposase of *CsuPLE1.1*. The ORF1 encodes GAG protein (related to viral structural protein), the ORF2 encodes AP (aspartic protease) protein, the ORF3 encodes a polyprotein including RT (reverse transcriptase), RH (RNase H) and IN (integrase). The blank triangles represent the LTRs (long terminal repeats).

Based on the flanking sequence of *CsuPLE1.1*, PCR with the primer pairs Flk-PBF (5′-TAACTAAGGTTCGCTGATGAC-3′) and Flk-PBR (5′-GATGCGCCTATCTATTTCG-3′) was carried out to obtain the *CsuPLE1.1* copy in different *C. suppressalis* individuals (Figure 1A). PCR was performed in a 10-μL reaction volume containing 30 ng gDNA, 0.4 mM of each dNTP, 1.5 mM of Mg^2+^, 0.2 μM of each primer, 1 μL of 10× LA PCR buffer (Mg^2+^ free) and 0.1 μL (5 U/μL) of *LA Taq*DNA polymerase (TaKaRa Biotechnology, Dalian, China). The amplification conditions were 3 min at 95 °C for initial denaturation, followed by 30 cycles of 30 s at 94 °C for denaturation, 30 s at 55 °C for annealing, and 3 min at 72 °C for extension, and a final elongation at 72 °C for 10 min. PCR products were purified with an AxyPrep DNA Gel Extraction kit (Axygen) and directly cloned into the pGEM-T Easy vector (Promega, Madison, WI, USA), and three clones of each PCR product were sequenced. Once nucleotide variations were detected in a clone, three more randomly selected clones of PCR product were sequenced to verify the variations. Multiple sequence alignments were performed with GeneDoc 2007. In each multiple sequence alignment group, the CsuPLE1-A01 was used as the ancestral copy for all variants (Dataset S1).

### 2.3. Determination of the Csu-Ty3 Copy at the Same Locus in Different Individuals

Based on the flanking sequence of *Csu-Ty3*, PCR with the primer pairs Flk-Ty3F (5′-ATCCATTGGCGTGCTGTTAT-3′) and Flk-Ty3R (5′-TACACAATAGAGCGTTCTGCTAC-3′) were used to get the *Csu-Ty3* copy in different *C. suppressalis* individuals. The *Csu-Ty3* intact sequence is quite long (~5000 bp), and therefore only a portion of the *Csu-Ty3* sequence (~1900 bp) was selected for further analysis. The primer pairs Flk-Ty3F (5′-ATCCATTGGCGTGCTGTTAT-3′) and Ty3inner-R (5′-CTGATGCACATCTTATACCGAAG-3′) were used to amplify a fragment from the *Csu-Ty3* copy from the same locus in different individuals (Figure 1B).

PCR was performed in a 25-μL reaction volume containing 50 ng gDNA, 0.4 mM of each dNTP, 1.5 mM of Mg^2+^, 0.2 μM of each primer, 2.5 μL of 10× LA PCR buffer (Mg^2+^ free) and 0.25 μL (5 U/μL) of *LA Taq*DNA polymerase. The amplification conditions were 3 min at 95 °C for initial denaturation, followed by 30 cycles of 30 s at 94 °C for denaturation, 30 s at 58 °C for annealing, and 5 min 20 s at 72 °C for extension, and a final elongation at 72 °C for 10 min. The resulting PCR products were purified and used as templates for the second round PCR. In the second round PCR, the final reaction volume and the reagent concentrations are the same as the first PCR. The second PCR amplification conditions were 3 min at 95 °C for initial denaturation, followed by 30 cycles of 30 s at 94 °C for denaturation, 30 s at 58.5 °C for annealing, and 2 min at 72 °C for extension, and a final elongation at 72 °C for 10 min. The resulting PCR fragments were also purified, cloned and sequenced as previously described. Multiple sequence alignments were performed with GeneDoc 2007. In each multiple sequence alignments group, the *Csu-Ty3*-A01 element was considered as the ancestral copy for all variants (Dataset S2).

### 2.4. Substitution, Deletion and Insertion Mutations Analyses

Based on the multiple sequence alignments, a mutation at a certain position may be observed many times. Unless otherwise specified, a mutation occurring multiple times at the same position has only been considered once. All these variants in each alignment were checked to ensure they were correct.

All the sequence elements from the host genome were cloned and sequenced in the same directions for comparability. Based on multiple sequence alignments, if a base in most sequence elements (more than 85%) was A, while in few sequence elements (less than 15%) was G, such case was recorded as, A mutated to G (i.e., A→G). All other base substitution cases in this work were recorded based on this criterion. Otherwise, the error rate of the Taq DNA polymerase used in this work is far less than 15%.

Single-base substitutions and multiple-base substitutions were analyzed separately. In the single-base substitutions, the transitions and transversions were recorded separately. In transitions, there are four patterns, i.e., A→G, G→A, T→C and C→T. In transversions, there are eight patterns, i.e., A→T, T→A, A→C, C→A, G→T, T→G, G→C and C→G. These 12 patterns of single-base substitutions were also recorded separately.

In the ORFs region of the two transposons, a single-base substitution can occur at position 1, 2 or 3 (pos1, pos2 or pos3) of a given codon. At each position, the numbers of single-base substitutions were recorded. Also in the corresponding amino acids and the single-base substitutions were sorted as synonymous, missense, nonsense and no-stop mutations. The numbers of these four patterns of mutations were recorded separately. Meanwhile, at each position of a codon, the numbers of different patterns of mutations were also recorded separately.

The numbers of multiple-base substitutions, deletions and insertions were also recorded and analyzed. There was a possibility that insertions in one sequence copy may be deletions in other elements. In order to ensure that the recorded data were comparable, based on the multiple sequence alignments in a certain position, if the base(s) existed in most elements (more than 85%) while in few elements (less than 15%) if did not exist, such a case was recorded as a base deletion, and vice versa for a base(s) insertion.

### 2.5. Statistical Analysis

For *CsuPLE1.1*, one individual copy was randomly selected from each of 21 field populations as one group. For *Csu-Ty3*, five individual *Csu-Ty3* elements were randomly picked from each of seven field populations and considered as one group. The statistical analyses were performed with SPSS (V13.0; Chicago, IL, USA).

When multiple-base substitutions, deletions and insertions were recorded, all 84 copies of *CsuPLE1.1* and 140 copies of *Csu-Ty3* elements were grouped together for multiple alignments because there were few multiple-base substitutions, deletions or insertions.

## 3. Results

### 3.1. Single-Base Substitution Mutations in CsuPLE1.1 Copies and Csu-Ty3 Elements

In this work, four groups were generated from 84 individual samples from *CsuPLE1.1* copies and four groups from the 140 individual samples from *Csu-Ty3* elements. Values were expressed as the mean of the four observed frequencies.

The summary of results for single-base substitution mutations for *CsuPLE1.1* and *Csu-Ty3* were in Supplementary Materials Dataset S3 and Dataset S4. In total, there were 62.75 single-base substitutions in the *CsuPLE1.1* copies, which consisted of 44.75 transition and 18.00 transversion substitutions. In the *Csu-Ty3* elements there were a total of 76.25 single-base substitutions consisting of 51.5 transition and 24.5 transversion substitutions (Table 1). In both *CsuPLE1.1* and *Csu-Ty3* elements, the transitions occurred much more frequently than transversions (*p* ≤ 0.01) (Table 1).

**Figure 2:**
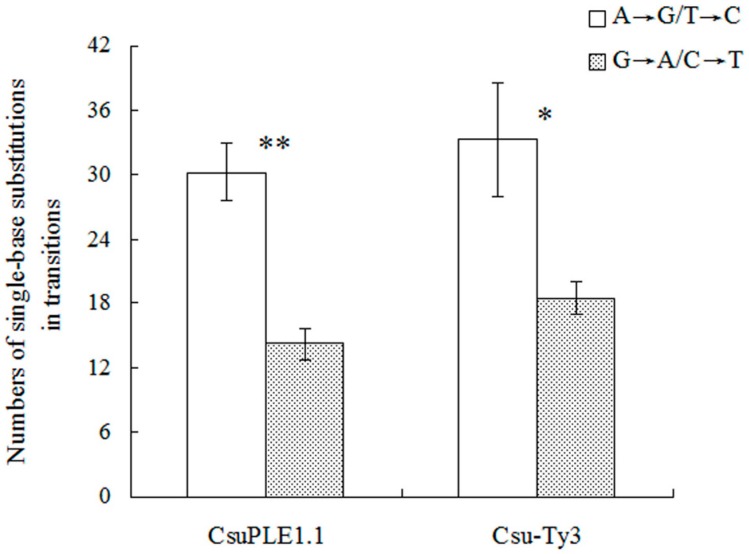
Single-base substitution patterns in transitions of *CsuPLE1.1* and *Csu-Ty3*. ** and * indicate significance at the 0.01 and 0.05 levels of probability, respectively. Independent-sample *T* Test by SPSS V13.0.

In these single-base substitutions, the most frequently occurring base change was A→G, in both *CsuPLE1.1* copies and *Csu-Ty3* elements. In *CsuPLE1.1* copies, there were 15.75 (mean) A→G substitutions, 14.5 T→C substitutions, 7.5 G→A substitutions and 7 C→T substitutions. In *Csu-Ty3* elements, there were 17.75 (mean) A→G substitutions, 15.5 T→C substitutions, 10.25 G→A substitutions and 8.25 C→T substitutions (Table 2). In transition substitutions of *CsuPLE1.1* copies, the sum of A:T→G:C was 30.25, while the sum of G:C→T:A was 14.25. The variation between them was significant (*p* ≤ 0.01). In *Csu-Ty3*, the sum of A:T→G:C and G:C→T:A was 33.25 and 18.5, respectively. The variation between them was also significant (*p* ≤ 0.05) (Figure 2).

Substitutions inside and outside the ORF were also determined. Transitions inside the ORF region, in both *CsuPLE1.1* and *Csu-Ty3*, occurred more frequently than transversions (*p* ≤ 0.01) (Figure S1). Similarly, the most frequently occurring base substitution was A→G, whilst the second most frequently occurring base change was T→C. In transitions, there were 11.25 (mean) A→G substitutions, 8 T→C substitutions, 6.5 G→A substitutions and 5.5 C→T substitutions in *CsuPLE1.1* copies and, there were 13.75 (mean) A→G substitutions, 11.75 T→C substitutions, 8.5 G→A substitutions, 6.75 C→T substitutions in *Csu-Ty3* elements. Similarly, the A→G/T→C substitutions occurred significantly more frequently than G→A/C→T (*p* ≤ 0.01 for *CsuPLE1.1*, *p* ≤ 0.05 for *Csu-Ty3*) (Figure S2). Additionally, the outside of ORF regions of *CsuPLE1.1* copies and *Csu-Ty3* elements were also analyzed. In *CsuPLE1.1* copies, outside of ORF, transitions occur more frequently than transversions (*p* ≤ 0.05). In *Csu-Ty3* elements, there is no significant difference between the transitions and transversions in this region (Figure S3).

**Figure 3:**
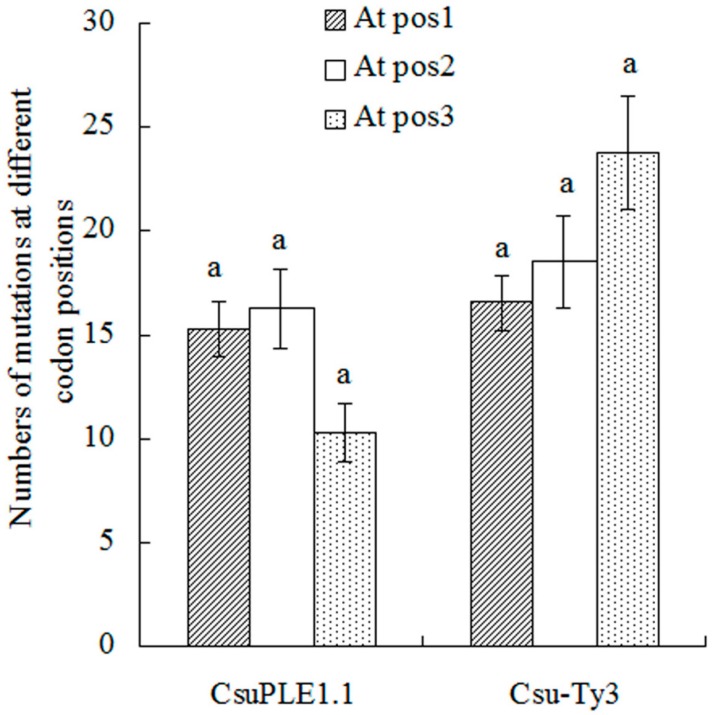
The single-base substitutions occurring at different positions of a codon. The data were analyzed using one-way ANOVA by SPSS V13.0. The average values of substitutions were separated using Tukey’s test. Within the same item in the abscissa, the same lowercase letters indicate there was no significant difference at the 0.05 level of probability.

In the ORF region, at each position of a given codon, the numbers of single-base substitutions were different. In *CsuPLE1.1* copies, there were 15.25, 16.25 and 10.25 single-base substitutions at pos1, pos2 and pos3, respectively. There were no significant differences among the three positions. In *Csu-Ty3* elements, there are 16.5, 18.5 and 23.75 single-base substitutions at pos1 (see Section 2.4), pos2 and pos3, respectively (Table S2). There were also no significant differences among the three positions (Figure 3).

**Figure 4:**
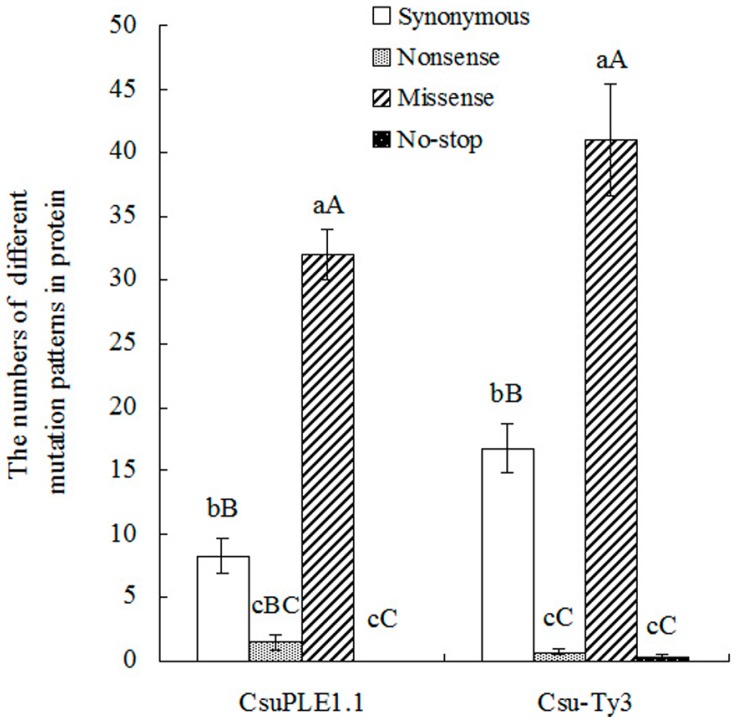
Different mutation patterns in proteins of *CsuPLE1.1* and *Csu-Ty3*. The data were analyzed using one-way ANOVA by SPSS V13.0. The average values of different mutation patterns were separated using Tukey’s test. Within the same item in the abscissa, different uppercase and lowercase letters indicate significance at the 0.01 and 0.05 levels of probability, respectively.

The numbers of four types of mutations were different, e.g., missense and synonymous mutations occurred frequently, while nonsense and no-stop mutations occurred less frequently (Figure 4).

### 3.2. Two-Base or More Substitution Mutations in CsuPLE1.1 Copies and Csu-Ty3 Elements

**Figure 5:**
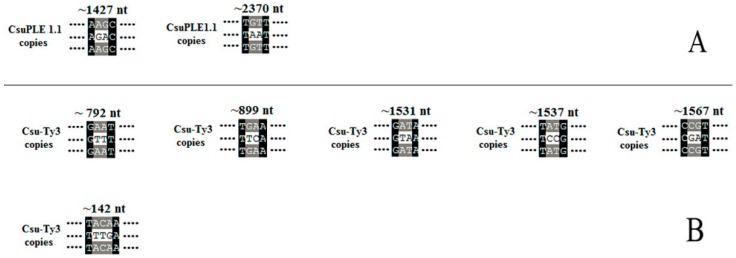
Two-base or more substitutions in *CsuPLE1.1* and *Csu-Ty3*. The location of each substitution in the sequence is indicated by Arabic numbers on the top. The omitted sequences are indicated by ellipses.

In *CsuPLE1.1* copies, there were only two 2-base substitutions, one AG→GA (inside the ORF region) and one GT→AA (outside the ORF region) (Figure 5A). In *Csu-Ty3* elements, there were five 2-base substitutions, one AA→TT, one GA→TC, one CG→GA, one AT→TA and one AT→CC. All of these five 2-base substitutions were inside the ORF regions. Additionally, there was only one 3-base substitution, ACA→TTG (Figure 5B).

### 3.3. Deletion and Insertion Mutations in CsuPLE1.1 Copies and Csu-Ty3 Elements

Both in *CsuPLE1.1* copies and *Csu-Ty3* elements, there were several multiple-base substitutions and indels (Table 3). In *CsuPLE1.1* copies, there were eight single-base deletions, five single-base insertions, five 2-base or more deletions and six 2-base or more insertions. In *Csu-Ty3* elements, there were eight single-base deletions, six single-base insertions, four 2-base or more base deletions and four 2-base or more base insertions. Indels that occurred inside the ORF region cause frame shifts or in-frame mutations.

## 4. Discussion

Both in *CsuPLE1.1* copies and *Csu-Ty3* elements, there are significant differences between the sum of A:T→G:C and the sum of G:C→T:A substitutions. However, in some studies, the A:T→G:C are less frequent than G:C→A:T substitutions. Genomes of bacteria are universally biased toward AT [14]. In the non-LTR retrotransposable element (*Helena*) and *Drosophila*, the frequencies of different nucleotide substitutions are not equal [6]. The greatest bias is in the high frequency of transition from G:C→A:T [6]. In the human genome, G:C→A:T is more frequent than A:T→G:C regardless of the background GC composition [5]. Studies demonstrate no relevance between GC composition and substitution bias [5,14]. The GC compositions of *CsuPLE1.1* copy and *Csu-Ty3* element are 35.9% and 37.3%, respectively. Thus, there might be no relevance between the low GC composition and the bias towards GC in *CsuPLE1.1* copies and *Csu-Ty3* elements.

Some researches indicated that DNA methylation in TEs was one of the methods of regulation or control by host [15]. Genome-wide analyses have revealed that DNA methylation is enriched in TEs and repeats [16,17,18]. The role of DNA methylation in TE silencing has been directly demonstrated using mutants of Arabidopsis. When DNA methylation is lost in the mutants of DNA methyltransferases or a chromatin remodeler DDM1 (decrease in DNA methylation 1), many of the TEs are de-repressed and mobilized [19,20]. Other researches showed that sequences with multiple suppressible mutations were very active when the transposons were unmethylated, but near normal when they were methylated [21,22]. Thus, more frequent A:T→G:C changes than G:C→A:T in *CsuPLE1.1* and *Csu-Ty3* may be related to the occurrence of transposon methylations.

It is generally assumed that the transition rate is higher than the transversion rate in animal genomes, as well as in the mitochondrial DNA [5,23,24]. In our study, transitions occur more frequently than transversions. This is additional evidence that universal bias occurs in favour of transitions over transversions. Purines and pyrimidines have a different size conformation, where purines have a bicyclic structure, and pyrimidines have a single ring structure. The process of transversion is probably more complicated than the process of transition and this may be one of the reasons that transitions are more frequent than transversions [25]. However, there is a case where transition–transversion bias is not universal. For example, in grasshopper pseudogenes, there is no significant difference between transition and transversion rates [26]. Therefore, occurrence of transition–transversion bias differs based on region of the genome. For example, genes (introns, exons, etc.), pseudogenes and transposable elements differ in the rate of spontaneous mutations probably due to the action of natural selection. Meanwhile, the spontaneous mutation rates and the natural selection pressures are probably different [6,25,26].

Many studies focusing on the patterns of nucleotide mutations in the genome are based on the genome sequencing data. Due to the technical drawbacks, there are often errors in sequencing results, which may cause errors in the study of mutation patterns and frequencies. Furthermore, some indels and substitution mutation patterns among different individuals were probably lost when the genomic DNA samples are taken from a specific geographic area. This situation will be more common in organisms that are widely distributed, but there is low gene exchange between field populations. Toshiyuki studied the substitution pattern at 10 loci in the telomeric region of the X chromosome for four species of *Drosophila melanogaster* subgroup [27]. Based on the sequencing results, the substitution patterns varied between different sequences, probably due to the differences between species [27]. In our work, based on the PCR and the sequencing, the nucleotide sequences are accurate. Thus, the nucleotide substitutions and indels of sequences in this work are very reliable. Besides, the sampling area is in a large scale in this work. Taken together, the substitution patterns widely represent the variation characteristics of transposon sequences between individuals of *C. suppressalis*. Few transposon sequences have great variations in individuals. This indicates that *C. suppressalis* individuals in different areas probably suffered various selective pressures.

## 5. Conclusions

The patterns of nucleotide substitution in these two types of transposons in *C. suppressalis* have the same characteristics. Mutations in *CsuPLE1.1* and *Csu-Ty3* sequences are biased towards GC. However, the mechanism underlying the occurrence of more frequent A:T→G:C changes than G:C→A:T related to the transposon’s activity or silencing needs further investigation.

## Figures and Tables

**Table 1:** The number of single-base substitutions in transitions and transversions.

Patterns		Numbers of Substitutions * (Means ± SE)
*CsuPLE1.1*	Transition	44.5 ± 3.52 ^A^
	Transversion	18.25 ± 1.44 ^B^
*Csu-Ty3*	Transition	51.75 ± 6.63 ^A^
	Transversion	24.5 ± 1.66 ^B^

* Data followed by different uppercase letters are significantly different at 0.01 probability level. Data were analyzed by one-way ANOVA (Tukey’s post hoc test) by SPSS V13.0.

**Table 2:** The number of single-base substitutions in transitions.

Transition Patterns		Numbers of Substitutions * (Means ± SE)
*CsuPLE1.1*	A→G	15.75 ± 1.7 ^aA^
	G→A	7.25 ± 0.95 ^bB^
	T→C	14.5 ± 1.32 ^aAB^
	C→T	7 ± 1.58 ^bB^
*Csu-Ty3*	A→G	17.75 ± 2.78 ^aA^
	G→A	10.25 ± 1.25 ^abA^
	T→C	15.5 ± 2.53 ^abA^
	C→T	8.25 ± 0.48 ^bA^

* Data followed by different uppercase and lowercase letters were significantly different at 0.01 and 0.05 probability levels. Data were analyzed by one-way ANOVA (Tukey’s post hoc test) by SPSS V13.0.

**Table 3:** One-base or more deletions and one-base or more insertions in *CsuPLE1.1* and *Csu-Ty3*.

Transposon	Region	1 Base Deletion	2 or more Base Deletion	1 Base Insertion	2 or More Base Insertion
*CsuPLE1.1*	Inside of the ORF region	1415_1417: one T deletion 1531_1533: one A deletion 1735_1737: one G deletion	750_761: 10 nt deletion 1530_1533: AA deletion 1606_1732: 125 nt deletion 2118_2128: 9 nt deletion	1532_1533: one A insertion	1798_1799: AGGTATA insertion
	Outside of the ORF region	49_51: one A deletion 50_52: one T deletion 77_79: one T deletion 556_558: one A deletion 2312_2314: one T deletion	79_91: 11 nt deletion	50_51: one A insertion 129_130: one A insertion 548_549: one G insertion 2313_2314: one T insertion	207_208: ACG insertion 539_540: CCTGCCT insertion 2313_2314: TT insertion 2313_2314: TTT insertion 2313_2314: TC insertion
*Csu-Ty3*	Inside of the ORF region	1133_1135: one G deletion 1389_1391: one G deletion 1397_1399: one T deletion 1646_1648: one A deletion 1810_1812: one T deletion	65_1029: 963 nt deletion 1539_1567: 27 nt deletion	272_273: one C insertion 555_556: one A insertion 576_577: one G insertion 1852_1853: one A insertion	896_897: TTCA insertion 1163_1164: TTAT insertion 1529_1530: 30 nt insertion
	Outside of the ORF region	47_49: one A deletion 128_130: one A deletion 729_731: one A deletion	696_701: CTTT deletion 695_701: TCTTT deletion	19_20: one A insertion 200_201: one T insertion	139_140: TGTGA insertion

The numbers indicate the position of a nucleotide in sequences. The form of “numbers_numbers” means that the deletions or insertions occurred between these two positions.

## Supplementary Materials

The following are available online at www.mdpi.com/2073-4425/7/10/72/s1, Table S1: longitude and latitude of sampling locations, Table S2: numbers of single-base substitutions at different positions of a codon, Figure S1-S2: the single-base transitions and transversions inside of ORF, Dataset S1 and S3: multiple sequence alignments of CsuPLE1.1 copies, Dataset S2 and S4: multiple sequence alignments of Csu-Ty3 copies.
