# Supplementary material for: Transition and Transversion Mutations Are Biased towards GC in Transposons of Chilo suppressalis (Lepidoptera: Pyralidae)
Source: Genes (Basel). 2016 Sep 24;7(10):72. doi: 10.3390/genes7100072 (PMC5083911; doi:10.3390/genes7100072)
Supplement: Supplementary file 1 [file genes-07-00072-s001.zip › genes-142308-supplementary/genes-142308-Supplementary file-tables and figures.docx]

Supplementary Materials: Transition and Transversion Mutationsare Biased towards GC in Transposons of *Chilo suppressalis*
(Lepidoptera: Pyralidae)

Guang-Hua Luo, Xiao-Huan Li, Zhao-Jun Han, Zhi-Chun Zhang,Qiong Yang, Hui-Fang Guo
and Ji-Chao Fang

**Table S1.** Longitude and latitude of sampling locations.

| **No.** | **Sampling location** | **Longitude (E)** | **Latitude (N)** |
| --- | --- | --- | --- |
| 1 | Gongzhuling (GZL) | 124:42:37 | 43:36:58 |
| 2 | Ganyu (GY) | 119:08:53 | 34:47:32 |
| 3 | Funan (FN) | 115:34:34 | 32:36:50 |
| 4 | Yizheng (YZ) | 119:14:09 | 32:31:50 |
| 5 | Hexian (HX) | 118:21:25 | 31:43:03 |
| 6 | Deyang (DeY) | 104:20:36 | 31:07:56 |
| 7 | Tongcheng (TC) | 116:53:25 | 30:57:23 |
| 8 | Jingzhou (JZ) | 112:20:29 | 30:16:48 |
| 9 | Qichun (QC) | 115:27:09 | 30:13:24 |
| 10 | Yinxian (YX) | 121:31:43 | 29:48:26 |
| 11 | Leshan (LS) | 103:41:51 | 29:31:18 |
| 12 | Jiangjin (JJ) | 106:15:54 | 29:08:28 |
| 13 | Xiangyin (XY) | 112:54:28 | 28:38:45 |
| 14 | Nanchang (NC) | 115:56:42 | 28:33:28 |
| 15 | Yongjia (YJ) | 120:48:00 | 28:02:30 |
| 16 | Shaoyang (SY) | 111:28:10 | 27:17:36 |
| 17 | Minhou (MH) | 119:03:54 | 26:13:58 |
| 18 | Ganxian (GX) | 115:02:03 | 25:56:10 |
| 19 | Yangshuo (YS) | 110:23:28 | 24:51:52 |
| 20 | Longhai (LH) | 117:50:06 | 24:27:15 |
| 21 | Guangning (GN) | 112:25:46 | 23:35:52 |

**Table S2.** Numbers of single-base substitutions at different positions of a codon.

| **Transposons** | **Position** | **Numbers of Substitutions *** |
| --- | --- | --- |
| CsuPLE1.1 | Pos 1 | 15.25 ± 1.25 a |
|  | Pos 2 | 16.25 ± 1.89 a |
|  | Pos 3 | 10.25 ± 1.38 a |
| Csu-Ty3 | Pos 1 | 16.5 ± 1.32 a |
|  | Pos 2 | 18.5 ± 2.22 a |
|  | Pos 3 | 23.75 ± 2.72 a |

***** Data followed by different lowercase letters mean significant difference at 0.05 probability level. Data were analyzed by One-way ANOVA (Tukey’s post hoc test) by SPSS V13.0.


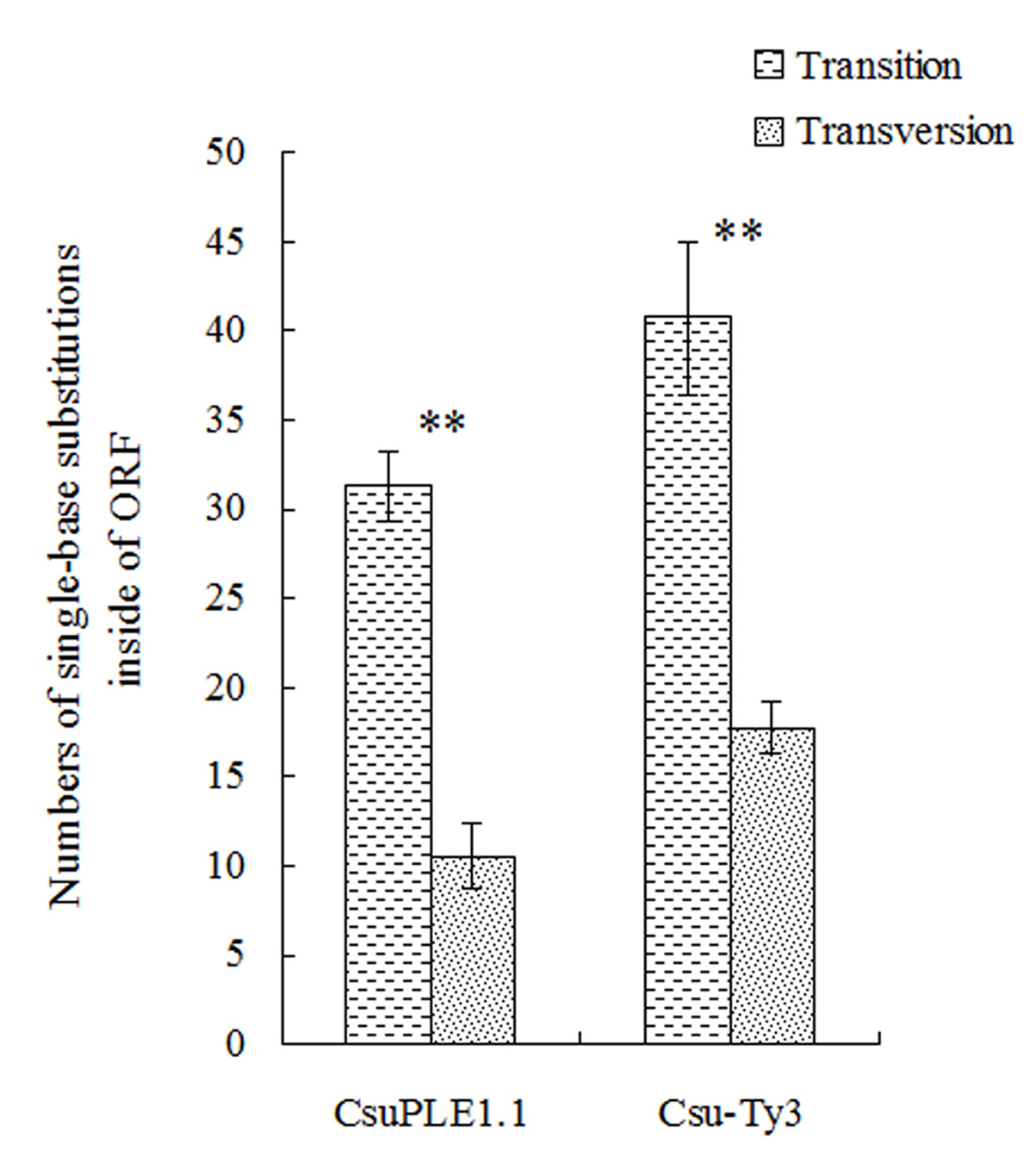


**Figure S1.** The single-base transitions and transversions inside of ORF. ** indicates significance at the 0.01 level of probability. Independent-Samples *T* Test by SPSS V13.0.


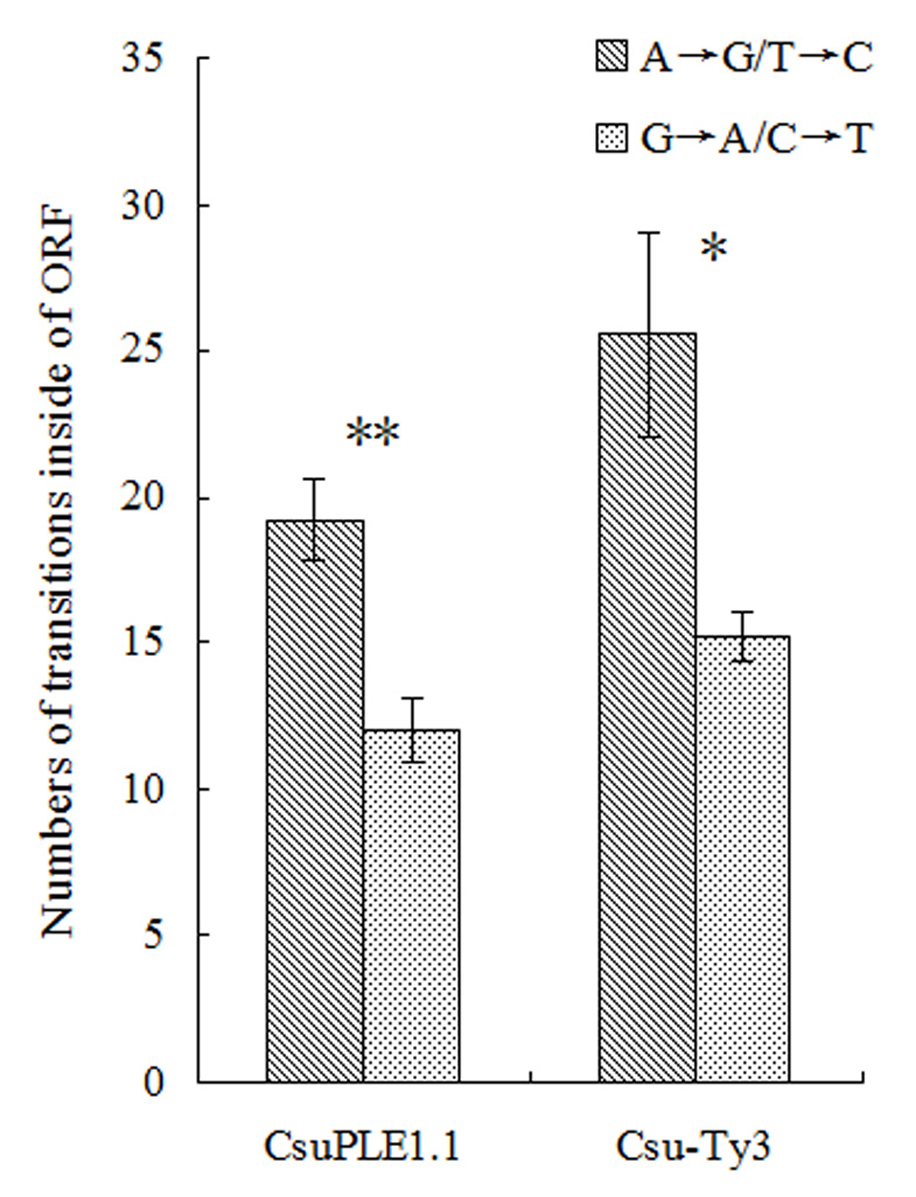


**Figure S2.** The single-base transitions inside of ORF. ** and * indicate significance at the 0.01 and 0.05 levels of probability, respectively. Independent-Samples T Test by SPSS V13.0.


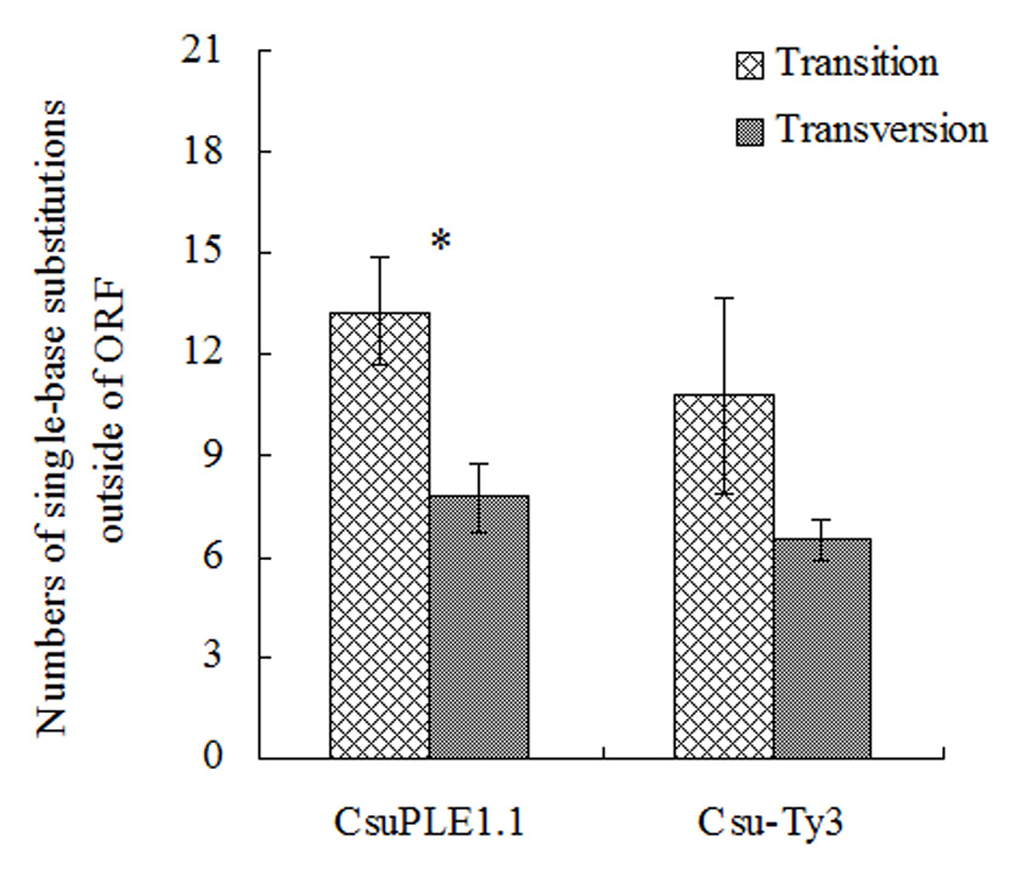


**Figure S3.** The single-base transitions outside of ORF. * indicates significance at the 0.05 level of probability. Independent-Samples T Test by SPSS V13.0.
