# Supplementary material for: Transition and Transversion Mutations Are Biased towards GC in Transposons of Chilo suppressalis (Lepidoptera: Pyralidae)
Source: Genes (Basel). 2016 Sep 24;7(10):72. doi: 10.3390/genes7100072 (PMC5083911; doi:10.3390/genes7100072)
Supplement: Supplementary file 1 [file genes-07-00072-s001.zip › genes-142308-supplementary/Supplementary file-Dataset S2.doc]

Dataset S2. Multiple sequence alignments of Csu-Ty3 copies

Group-1:

Group-2:

Group-3:

Group-4:
